# Digital stress perception among German hospital nurses and associations with health-oriented leadership, emotional exhaustion and work-privacy conflict: a cross-sectional study

**DOI:** 10.1186/s12912-024-01825-z

**Published:** 2024-03-28

**Authors:** Jessica Kräft, Tanja Wirth, Volker Harth, Stefanie Mache

**Affiliations:** Institute for Occupational and Maritime Medicine (ZfAM), University Medical Center Hamburg-Eppendorf (UKE), 20459 Hamburg, Germany

**Keywords:** Technostress, Nursing staff, Health, Health-oriented leadership

## Abstract

**Background:**

The use of digital information and communication technologies (ICT) can be accompanied by increased technostress for nursing staff, which in turn can be associated with health consequences. In addition, the use-related constant accessibility through ICT can have a negative impact on health-related recovery and regeneration phases. Health-promoting behaviors of supervisors can influence health complaints and conflicts between employees’ work and private lives. The present study investigates whether there is a corresponding relationship between digital stressors (technostressors) as well as health-oriented leadership and health outcomes among nurses.

**Methods:**

In a quantitative online survey, hospital nursing staff (*n* = 243) was asked about techno-invasion, social environment, emotional exhaustion, work-privacy conflict and on the supervisors’ health-oriented staff-care dimensions awareness, value of health and health-oriented leadership behavior (HoL: awareness, value of health and health behavior). The associations of technostress, HoL and health outcomes were tested using regression analyses and performing a correlation.

**Results:**

Significant positive associations between techno-invasion and health outcomes had been found. Social environment was not (positively) significantly related to either emotional exhaustion or work-privacy conflict. Health-oriented leadership moderated the association between social environment and work-privacy conflict.

**Conclusions:**

The results confirm the relevance of measures to reduce technostress and the importance of health-oriented leadership as a health-promoting resource. For practice, offers should be implemented for a balanced work and personal life of the nursing staff as well as establishing competence trainings for supervisors to learn and implement health-promoting behaviors. When technology use can’t be reduced, options could be created to ensure that nurses’ work and private lives become more balanced. These could represent mindfulness practices.

## Background

Nursing staff is exposed to particular work-related stress factors [1, 2]. The high work intensity in medical care, which is characterized by long working hours, shift work and a multitude of tasks can lead to emotional exhaustion and other negative health consequences [3–9]. The current technologization of medical care structures and the already widespread use of information and communication technology (ICT) has the potential to further increase the demands on the nursing profession [10, 11]. The resulting strenuous technostress can lead to negative consequences for the individual [12–16]. Explicit individual stress consequences include subjective work stress experience, emotional exhaustion, depression, the increase of stress hormones and reduced work productivity [4, 5, 17–21]. Technostress is defined as stress caused by the use and omnipresence of digital technologies [17, 22]. Ragu-Nathan et al. describe five indicators of technostress: techno-overload, techno-insecurity, techno-invasion, techno-uncertainty and techno-complexity [22]. Building on this, Riedl et al. developed a more differentiated instrument that measures technostress in the work context [23].

The theoretical embedding of this study is ensued by the occupational psychological stress model according to Bamberg [24]. Within this model, resources, condition-related stressors, person-related risk factors, coping and evaluation mechanisms, and stress consequences are included, which interact in numerous ways. The technostressors examined in this study, techno-invasion and social environment, are classified as condition-related stressors as they occur in the context of working conditions and the work environment. Further elements of the model are person-related resources (abilities and characteristics of the individual such as self-efficacy) and condition-related resources (from the environment of the individual such as the work environment) [24]. The influence of health-oriented leadership behavior represents a condition-related resource. Stress consequences according to this model can be divided into somatic, cognitive-emotional and behavioral. Emotional exhaustion represents a cognitive-emotional consequence of stress that also has long-term somatic effects. The conflict between work and private life, which not only affects the individual, is a social and behavioral consequence of stress.

### Current state of research

A study of the GSA region (Germany, Switzerland, Austria region) shows that the two technostressors techno-invasion and social environment in particular cause the highest levels of stress perception among participants [13]. Usage-related constant accessibility (techno-invasion) through ICT has negative effects on work-life balance [13]. Work-related smartphone use in work-free time can then lead to unwanted, social pressure to answer emails and messenger messages directly and has a negative impact on mental health [13, 25]. An expectation to be available after work is created. However, a healthy work-life balance plays a crucial role in recovering from work stress [25, 26]. If work-related stressors have a negative impact on family and personal life, this can lead to work-privacy conflict (WPC). WPC is defined as an interrole conflict that exists due to interfering demands and strains created by job with private responsibilities [27]. In some cases, studies have already shown that technostress can lead to WPC [15, 28, 29].

As a further health consequence of technostress, the authors Riedl et al. show that the higher the perceived technostress, the higher the emotional exhaustion [13]. To overcome the challenges of technostress in the work context, the literature clearly points to the role of the supervisor. The study by Montano et al. shows that leadership has a positive effect on the health of employees [30]. This effect is also supported in other studies [10, 12, 30, 31]. Bauwens et al. also show in their study that supervisors can act as role models to set stricter boundaries between work and private life and use participative decision-making processes to determine whether, for example, e-mails are only answered at certain times of the day [12, 30]. A leadership-related reduction of stress levels and an improvement in employees’ health complaints has also been demonstrated in other studies [30, 32, 33].

A study also shows that employees who perceive their supervisors as health-oriented take more care of their own health and thus have fewer health complaints, less psychological irritation and fewer conflicts between work and private life [34]. Health-related behaviors of the supervisor influence the health of employees and their perception of technostress [10, 30, 35]. The integrative leadership concept that measures health-related behaviors of supervisors is summarized under the Health-oriented-Leadership (HoL) approach by Franke et al. The approach defines four central aspects that characterize health-specific leadership behaviors: Health valence, health-related mindfulness, health behaviors, and lifestyle [36].

Another study examined the HoL approach and analyzed whether there is a connection between HoL and the health of prospective teachers [31]. According to this study, HoL has a positive effect on the health of teachers and reduces their experience of stress [31]. The target group by Arnold et al. has comparable parameters to the present target group of nursing staff as their day-to-day work is also characterized by a large number of stressors, a high sickness rate and a shortage of staff. Regardless of the widely assumed direct correlation between leadership and employees’ health, leadership is increasingly recognized as a moderating influencing factor in empirical research when examining employee health [12, 30, 37].

### Aim of the study

Including the studies reviewed as part of the technostress research, only a few studies were identified that consider the association of leadership and reduced negative consequences of technology [12, 30]. Furthermore, only an insufficient study base was identified that examines the relationship between HoL and health outcomes due to technostressors among nursing staff. Previous studies call for proactive research on effective interventions to reduce technostress [37–39]. Therefore, the rationale for this study is to provide empirical evidence on technostress perception and associations to health-oriented leadership among nursing staff and to contribute to filling the aforementioned research gaps. Accordingly, the aim of the present study is to examine the relationship between the health outcomes, emotional exhaustion and WPC, with the technostressors techno-invasion and social environment among hospital nursing staff. In addition, this study aims to investigate the role of HoL between the occurrence of the two technostressors (techno-invasion and social environment) and emotional exhaustion and WPC.

### Hypotheses

The technostressor techno-invasion is associated with health consequences such as emotional exhaustion [12, 23, 40, 41]. Social environment caused the most stress among the participants in the GSA region study [13]. The following hypotheses are derived from this based on the occupational psychology stress model:

#### H1a-b

There is a positive relationship between (a) techno-invasion as well as (b) social environment and nurses’ emotional exhaustion.

#### H1c-d

There is a positive relationship between (c) techno-invasion as well as (d) social environment and nurses’ WPC.

The importance of leadership as a positive influencing factor on employee health has been demonstrated in several studies [10, 12, 30, 31]. The following hypotheses are derived from this:

#### H2a-b

The less HoL is available, (a) the more emotional exhaustion is felt and (b) the greater the WPC is felt.

Bauwens et al. also examined whether the relationships between technostress, emotional exhaustion, and the quality of care provided were moderated by leadership behavior. In this study, the influence of techno-invasion decreased with certain leadership behaviors [12]. Therefore, a moderation effect of HoL on the relationship between technostressors and health outcomes is hypothesized:

#### H3a-b

A higher degree of HoL reduces (a) the positive relationship between techno-invasion and perceived emotional exhaustion and (b) the positive relationship between techno-invasion and perceived WPC.

#### H3c-d

A higher degree of HoL decreases (c) the positive association between social environment and perceived emotional exhaustion and (d) the positive association between social environment and perceived WPC.

## Methods

The present quantitative cross-sectional study took place in the form of an online survey during the recruitment period April 2023 to June 2023. The requirements for participation in the study were at least one year of employment as a nurse in inpatient acute care at a German hospital, with a minimum working time of 10 h per week. Sampling method was probability sampling: from January 2023 to March 2023, a recruitment register was created that contained an in-depth listing of clinics in Germany. The register was based on the website www.kliniken.de and included all acute care hospitals from Schleswig-Holstein, Hamburg, North Rhine-Westphalia, Hesse, Thuringia, Saxony, Baden-Württemberg, and Bavaria that were listed on the website. The federal states were purposely selected to cover the northern, eastern, southern and western regions of Germany. Rehabilitation clinics were excluded, because the job profile of nursing staffs differs significantly from that of nursing staff in acute care in hospitals. The final registry included 763 identified acute care hospitals, which were contacted via e-mails to nursing management with an information flyer about the study objective, procedure, and a link to the survey.

The online questionnaire developed for the survey with standardized items was created using the online survey tool *LamaPoll*. The survey instrument consisted of five sections: (A) demographic data, (B) questions on technology use based on frequency of use and type of technology used, (C) questions on the technostressors techno-invasion and social environment, (D) questions on HoL, and (E) questions on the health outcomes emotional exhaustion and WPC.

### Survey instrument

Technology use was queried via two questions. One question covered the work-related use of communication technologies and was, “Which communication channels do you use for work and how many hours did you use these communication channels on average per day in the last month?” The second question covered the work-related use of communication technologies outside of working hours and was, “Which communication channels do you use to communicate with your employer/colleagues on work-related matters outside of your working hours and how many hours on average did you use these communication channels per day in the last month?” An answer could be given for both questions in the range from 0 to ten hours per day.

One subscale of the German version of the Digital Stressors Scale was used to measure the technostressor social environment [23]. It comprises five items which are rated on a seven-point Likert scale (1="do not agree at all” to 7="agree completely”). An example item is “Too much time gets lost at work because of irrelevant communication with other people on social media.“, α = 0.78). The scale value is derived from the unweighted mean of the five underlying items.

The technostressor techno-invasion is derived from Ragu-Nathan’s Technostress Creators Scale [22]. The subscale contains five items, which are rated on a five-point Likert scale (1="strongly disagree” to 5="strongly agree”) [22]. An example item is “I feel my personal life is being invaded by this technology.“, α = 0.80). The scale score is the unweighted mean of the five underlying items. For the present study, the five items were translated into German. This was done following the German translation by the authors Gimpel et al [41].

HoL was assessed with the subscale staff-care for external assessment by employees of the HoL instrument of Franke et al. [36]. The subscale comprised a total of 22 items, each of which is answered on a five-point Likert scale (1="strongly disagree” to 5="strongly agree”). Six items capture health awareness (α = 0.86); an example item is “My supervisor often doesn’t realize until it’s too late that she/he has put too much on me.” Thirteen items cover health behavior of which ten items capture health behavior in the workplace (for example, “My supervisor ensures that my stresses are reduced by promoting positive interactions with each other.“, α = 0.87). Three items capture value of health, for example, “My supervisor tries to be a role model for me in terms of health,” α = 0.85). The scale values are derived from the unweighted mean values of the items.

Emotional exhaustion was measured using the six items of the German version of the subscale personal burnout from the Copenhagen Burnout Inventory (CBI) [42]. The items were answered on a five-point Likert scale (1="never/almost never” to 5="always”). Internal consistency is reported as α = 0.87; a sample item from the subscale is “How often are you physically exhausted?” [43].

The five items on the WPC originated from the Work-Family Conflict Scale by Netemeyer et al. and was further developed for the Copenhagen Psychosocial Questionnaire (COPSOQ) [27]. The five items (for example, “The time my work takes up makes it difficult to meet family or personal obligations.“) were recorded on a seven-point Likert scale (1="strongly disagree” to 7="strongly agree,” α = 0.91) [44].

The complete questionnaire was pretested for content validity and comprehensibility.

### Bias

Since stress perception differs according to individual factors such as gender, age and educational background whereas health status and psychological distress are influenced by age and gender, these were included as control variables in this study [45].

### Study size

According to the Federal Statistical Office, there are currently 486.100 registered nurses working in the inpatient sector in Germany [46]. With this population, assuming a confidence level of 90%, an error range of e = 0.05, and a standard deviation of *p* =.5, the required sample size for statistical analysis is *n* = 273.

### Statistical analysis

The analysis was performed using IBM SPSS software (version 29). Firstly, the questionnaire data was checked for plausibility, outliers, and missing values. The resulting sample was used to describe the demographic characteristics of the study participants and to calculate the relative and absolute frequencies for categorical variables. Mean (M) and standard deviation (SD) were calculated for each variable. The reliability parameter Cronbach’s alpha (α) was calculated for the item and scale analyses. In addition, a correlation analysis was performed according to the Pearson correlation coefficient. For the technology use data, the hours of use reported were divided into four categories for better presentation. Each category comprised five-time intervals.

To test hypotheses H1a-d as well as H2a-b, two linear regression analyses were conducted. For the first linear regression model the technostressors techno-invasion and social environment were included as predictors. Emotional exhaustion was used as the criterion variable. For the second regression model the technostressors techno-invasion and social environment were also included as predictors and WPC served as the criterion variable. Gender and age served as control variables in both models. For this purpose, age and gender were transformed into dummy variables for the analysis.

All data checks required for regression modeling were performed in advance (equality of variances, multicollinearity, homoscedasticity of data, normal distribution of residuals). The statistical significance level was set at *p* <.05.

To answer H3a-d, four interaction terms with the technostressors and health outcomes were formed in a moderation analysis. To further illustrate a significant interaction effect between HoL, social environment, and perceived WPC, the data set was divided into two subgroups. The division was based on a median split of HoL, following the test manual on HoL. Values of a median ≥ 2.8 indicate high levels of HoL, values of a median < 2.8 indicate low levels of HoL.

## Results

### Descriptive analysis

The survey website was visited 413 times. Complete data sets formed *n* = 244, of which *n* = 5 data sets did not meet the requirements of the study. In total 239 online surveys were included in the data analysis. Most participants came from clinics in the federal state of Bavaria (47.3%), followed by North Rhine-Westphalia (27.2%). Most of the clinics were publicly funded (70.3%).

The study population consisted mainly of female nurses (73.2%). Most participants were in the age groups 40–49 years (30.1%), the age group up to 19 years was not represented (0%). Table 1 shows the socio-demographic characteristics of the study population.

**Table 1:** Description of the study population

Variable	n (%)^1^
**Gender**	
Female	175 (73.2)
Male	63 (26.2)
Diverse	1 (0.4)
**Age**	
≤ 19 years	0 (0)
20–29 years	34 (14.2)
30–39 years	49 (20.5)
40–49 years	72 (30.1)
50–59 years	67 (28.0)
≥ 60 years	17 (7.1)
**Professional qualification**	
General Nurses	212 (88.7)
Pediatric Nurses	19 (7.9)
Geriatric Nurses	6 (2.5)
Nursing assistants	2 (0.8)
**Shift** ^2^	
Day shift (weekdays)	202 (84.5)
Night shift (weekdays)	102 (42.7)
Day or Night shift (Weekend)	160 (66.9)
**Extent of employment**	
Full-time (≥ 35 h)	184 (77.0)
Part-time (15–34 h)	52 (21.8)
Part-time (< 15 h)	3 (1.3)
**Federal state**	
Bavaria	113 (47.3)
North Rhine-Westphalia	65 (27.2)
Hesse	20 (8.4)
Hamburg	12 (5.0)
Other	39 (< 5)
**Number of beds**	
600 or more	95 (39.7)
300–599 beds	60 (25.1)
Up to 299 beds	77 (32.2)
Not specified	7 (2.9)
**Hospital carrier**	
Public	168 (70.3)
Free	45 (18.8)
Private	19 (7.9)

^1^*n* = 239; ^2^multiple choice

Participants indicated e-mails (*n* = 221) and phone calls (*n* = 226) as the most frequent work-related communication channels. Calls (*n* = 153) were most frequently used for communication outside of working hours with the employer/colleagues. A small number of participants, 41 people (16.6%), indicated other work-related communication channels. These included face-to-face meetings, online conferencing tools (MS Teams), fax and letters. Table 2 shows the work-related use of technology in more detail, based on the number of hours per day surveyed in the last month.

**Table 2:** Presentation of work-related technology use

Variable	n (%)^1^					
**Technology use** **– work-related**	Min.-Max. (hours)	No use	0.5–2.5^2^	3.0–5.0	5.5–7.5	8.0–10.0
E-Mails	0–10	18 (7.5)	169 (70.7)	40 (16.8)	8 (3.3)	4 (1.6)
Messenger-messages	0–10	130 (54.4)	97 (40.5)	5 (2.1)	6 (2.4)	1 (0.4)
SMS	0–2.0	229 (95.8)	10 (4.2)	-	-	-
Phone calls	0–10	11 (4.6)	126 (52.6)	63 (26.4)	23 (10.5)	14 (5.7)
Other	0–8	198 (82.8)	30 (12.6)	8 (3.3)	1 (0.4)	2 (0.8)
**Technology use** **-communication outside working hours**						
E-Mails	0–5	139 (58.2)	89 (37.4)	9 (3.8)	-	-
Messenger-messages	0-9.5	99 (41.4)	126 (52.7)	8 (3.3)	3 (1.2)	1 (0.4)
SMS	0-3.5	229 (95.8)	9 (3.8)	1 (0.4)	-	-
Phone calls	0–9.0	86 (36.0)	140 (58.7)	8 (3.3)	4 (1.6)	1 (0.4)
Other	0-3.5	234 (97.9)	4 (1.6)	1 (0.4)	-	-

^1^*n* = 239

^2^in hours (half hour apart)

Table 3 shows the characteristics of the main study variables. Reliability was confirmed for all scales (α > 0.7). The technostressor techno-invasion showed floor effects, as there was no differentiability in 27.2% (*n* = 65) between the participants (SD = 0%).

**Table 3:** Characteristics of the main study variables

Variables	Mean	SD	Range	Min	Max	α
Social environment	3.57	1.52	1–7	1	7	0.83
Techno-invasion	2.02	0.97	1–5	1	5	0.80
Emotional exhaustion	2.98	0.78	1–5	1	5	0.91
Work-privacy conflict	4.22	1.54	1–7	1	7	0.93
Health-oriented leadership	2.77	0.94	1–5	1	5	0.96

*Note* α = Cronbach’s Alpha

As shown in Table 4, the correlation analysis showed significant positive correlations between the technostressors techno-invasion and social environment as well as perceived emotional exhaustion (*r* =.310, *p* <.001; *r* =.256, *p* <.001) and perceived WPC (*r* =.408, *p* <.001; *r* =.350, *p* <.001). A significant negative correlation was found between HoL and perceived emotional exhaustion (*r* = −.274, *p* <.001). There was also a significant negative correlation between HoL and perceived WPC (*r* = −.366, *p* <.001).

**Table 4:** Correlation coefficients for the main study variables

	Variables	1	2	3	4	5
1	Techno-invasion	-				
2	Social environment	0.596***	-			
3	Work-privacy conflict	0.408***	0.350***	-		
4	Emotional exhaustion	0.310***	0.256***	0.733***	-	
5	Health-oriented leadership	− 0.092	− 0.174	− 0.366***	− 0.274***	-

Pearson correlation coefficient: *p <.05; **p <.01; ***p <.001

Table 5 shows the results of the regression analysis with emotional exhaustion as the dependent variable. The model as a whole was significant (F_(9,229)_ = 6.591, *p* <.001). For the test of H1a, the T-test for the regression coefficient of techno-invasion (β = 0.246, *p* =.001) was significant. For H1b, the T-test for the regression coefficient of social environment (β = 0.064, *p* =.401) was not significant. Age was not significantly associated with emotional exhaustion. There was a significant effect of female gender compared to male gender (β = 0.195, *p* =.001). The model had a mean goodness of fit with an R^2^ = 0.21 (corrected R^2^ = 0.18). With regard to hypotheses H1a-b, hypothesis H1a can be accepted.

**Table 5:** Regression analysis, dependent variable emotional exhaustion

Coefficients	b	SE	β***	T	p	95% CI
Constant	2.779	0.224		12.425	**< 0.001**	2.338; 3.220
Techno-invasion	0.179	0.060	0.246	3.312	**0.001**	0.080; 0.315
Social environment	0.032	0.039	0.064	0.842	0.401	− 0.043; 0.108
Health-oriented leadership	− 0.181	0.050	− 0.219	− 0.3642	**< 0.001**	− 0.279; − 0.083
Age (≥ 60 years)^1^	− 0.152	0.194	− 0.050	− 0.783	0.435	− 0.534; 0.230
Age (40–49 years)^1^	− 0.104	0.121	− 0.062	− 0.859	0.391	− 0.343; 0.135
Age (30–39 years)^1^	− 0.101	0.133	− 0.053	− 0.757	0.450	− 0.364; 0.162
Age (20–29 years)^1^	0.030	0.151	0.013	0.196	0.845	− 0.268; 0.327
Gender^2^ (female)	0.342	0.105	0.195	3.249	**0.001**	0.135; 0.550

*n* = 238; *R*^2^ = 0.206; corr. *R*^2^ = 0.175; F(9,229) = 6.591; *p* <.001

^1^in reference to age group 50–59 years; ^2^in reference to the male gender

The results of the regression analysis with WPC as the dependent variable can be found in Table 6. The model was significant overall (F_(9,229)_ = 10.710, *p* <.001). For H1c, the T-test for the regression coefficient of techno-invasion (β = 0.315, *p* <.001) was significant. For H1d, the T-test for the regression coefficient of social environment (β = 0.102, *p* =.152) was not significant. Age and gender each had no significant effect on WPC. The model had a high goodness of fit with an R^2^ = 0.30 (corrected R^2^ = 0.27) [47, 48]. With regard to hypotheses H1c-d, hypothesis H1c can be accepted.

For hypotheses H2a-b, whether HoL is related to health outcomes, a significant negative relationship was found between HoL (β = − 0.219, *p* <.001) and emotional exhaustion (Table 5). The relationship between HoL and perceived WPC was also significant (β = − 0.309, *p* <.001, Table 6). Hypotheses H2a-b can both be accepted.

**Table 6:** Regression analysis, dependent variable WPC

Coefficients	b	SE	β***	T	p	95% CI
Constant	4.024	0.418		9.623	**< 0.001**	3.200; 4.848
Techno-invasion	0.502	0.111	0.315	4.503	**< 0.001**	0.282; 0.721
Social environment	0.103	0.072	0.102	1.437	0.152	− 0.038; 0.245
Health-oriented leadership	− 0.507	0.093	− 0.309	-5.447	**< 0.001**	− 0.690; − 0.323
Age (≥ 60 years)^1^	0.011	0.363	0.002	0.032	0.975	− 0.703; 0.726
Age (40–49 years)^1^	− 0.151	0.226	− 0.045	− 0.668	0.505	− 0.597; 0.295
Age (30–39 years)^1^	0.097	0.249	0.025	0.387	0.387	− 0.395; 0.588
Age (20–29 years)^1^	0.133	0.282	0.030	0.473	0.473	− 0.423; 0.690
Gender^2^ (female)	0.306	0.197	0.088	0.1553	0.122	− 0.082; 0.694

*n* = 238; *R*^2^ = 0.296; corr. *R*^2^ = 0.269; F(9,229) = 10.710; *p* <.001

^1^in reference to age group 50–59 years; ^2^in reference to the male gender

A moderation analysis was conducted for hypotheses H3a-d. The results of the moderation analysis showed a significant moderation effect (ß= 0.449, *p* =.021) between HoL and social environment on perceived WPC. The overall model was significant (F_(2,235)_ = 24.170, *p* <.001). The model had a mean goodness of fit with an R^2^ = 0.24 (corrected R^2^ = 0.23). The relationship between social environment and perceived WPC is therefore moderated by HoL. Hypothesis H3d can therefore be accepted.

The subgroup analysis using the median split showed a stronger relationship between social environment and perceived WPC with low health-oriented leadership (ß = 0.200, *p* =.018) than with high HoL (ß = 0.463, *p* <.001).

## Discussion

This study investigates the relationship between technostressors, HoL and nurses’ health. The study results confirm the assumption of a connection between HoL and health outcomes. In addition, the correlation between techno-invasion and emotional exhaustion as well as WPC is confirmed.

### Descriptive analysis of technology use

With regard to context-related technology stress research, the present results show that technology stress is also perceived in the nursing profession due to ICT [49]. With regard to technology use for both work-related communication and communication with their employer/colleagues outside of working hours, the majority of participants stated they used e-mails and phone calls most frequently. This is consistent with previous studies in which e-mails and phone calls were also the most frequently used means of communication and interaction [16, 41]. Previous studies have also shown that the use of e-mails and telephone in particular is associated with stress [13]. Medical software such as electronic patient records or decision support systems were not included in this study, but are increasingly being used in hospitals [50]. It can therefore be assumed that the perception of technostress will continue to increase with increasing technologization.

### Relationships between technostressors and health outcomes

The significant association between techno-invasion and health outcomes is consistent with previous studies [12–15]. In particular, the association with emotional exhaustion is consistent with the reviewed literature [40, 41]. Despite the significant association with health outcomes, techno-invasion shows floor effects. It can be assumed that the present sample has less of a feeling of always being available everywhere and that the boundary between work and private life is not blurred. This is consistent with the results of a study where techno-invasion had the lowest mean value [41].

In the regression models of this study, the technostressor social environment was found to be a non-significant predictor of health outcomes. However, in the present study, the technostressor social environment caused a greater perception of stress than techno-invasion, which in turn is consistent with the results of Riedl et al. where social environment caused the most stress among the participants [13].

### Relationship between health-oriented leadership and health outcomes

The results for answering hypothesis 2a-b show that HoL, in particular the components awareness, value of health and health behavior are directly and negatively related to emotional exhaustion and WPC. The importance of HoL as a resource for employee health is thus consistent with previous studies [31, 34, 35]. The results also support the findings of the authors Horstmann et al. [38]. A health-oriented management approach can address the central challenges in the nursing profession in order to support employees in terms of their health and to emotionally bind them to the hospital [38].

The moderation effect postulated in H3a-d between HoL, technostressors and health outcomes can only be assumed for WPC. HoL is therefore not only directly related to health outcomes, but also indirectly influences the relationship between social environment and the WPC. However, the subgroup analysis shows that the technostressor is more strongly related to WPC when HoL is low. The correlation was weaker when the level of HoL was high. This contradicts the presumed assumption that leadership reduces this correlation, but is in line with the unexpected results of Bauwens et al. [12]. According to their results, leadership increases the effect of professional overload on the quality of care, which is mediated by emotional exhaustion. This is again consistent with the findings of Kim et al. who argue that overworked employees prefer less committed behaviors from their supervisors [51]. Health-related behaviors of the supervisor and the resulting measures could therefore be perceived by employees as an additional burden and increase existing stress or pressure [12, 51].

### Research and practical implications

Future research should examine the results in longitudinal studies in order to make statements about causal relationships and to confirm the direction of effect of the relationships found. In this context, it would be interesting to include other contextual factors in the model, such as the person-related resources like nurses´ own coping mechanisms or health literacy and work-related rules for dealing with technology.

In further studies, it is important to increasingly survey younger nursing staff on the topic of technostress. This target group will increasingly work with technology, which means that not only ICT, but also cleaning robots, AI-based decision support systems or electronic patient records will be used in hospitals [2, 50]. With regard to ICT, the communication strands should be considered so that more time is available for patient care.

In addition to further research on technostress, one should take care to design, especially online questionnaires or surveys, in a way that causes little to none technostress (e.g. through good applicability, usability, easy readability). As already mentioned in the introduction of this study, technostress is stress caused by an inability to cope with the demands of technology usage [17, 22]. Therefore, it is important to avoid possible bias when developing the questionnaire.

With regard to HoL, this study only provides an external assessment of the nursing staff towards their respective supervisor. Observational studies or qualitative interviews could be used to determine whether health-related behaviors are actually practiced in everyday clinical practice.

In addition, the technostressor social environment should be given greater consideration. It is most pronounced in the review study by Riedl et al. and is also higher than techno-invasion in the present study [13]. However, no significant association with health outcomes was found. It should be noted that this technostressor has only recently been included in technostress research and should therefore be validated in further studies [52].

Further studies could also focus more on the relationship between work-related use of technology, WPC and working hours of nursing staff. In this study, most participants worked full-time. Further research could focus on whether working part-time leads to less work-related use of technology and therefore less WPC.

With regard to practical implications, the study results indicate a need for behavioral and structural measures of occupational health promotion for nursing staff. Structural approaches should establish rules that enable work-related use of technology while maintaining a healthy work-life balance. If it is not possible to reduce technology use, options could be created to ensure that nurses’ work and private lives become more balanced. These could represent mindfulness practices [53, 54]. Practicing mindfulness as a cognitive–emotional segmentation strategy can help employees to set boundaries and increase detachment. In this context, mindfulness training has been shown to reduce psychological work-life conflict and enhance work-life balance [53] and could therefore be an additional behavioral prevention measure. Another starting point is the technology itself. One could try to increase satisfaction with ICT. One approach to this can be found in the study by Pasini et al., who developed and tested a game-based training program to cope with technostress [55].

The condition-related resource of HoL could be expanded through behavioral measures. Managers can learn health-promoting behaviors through skills training and implement them when dealing with employees. Caregivers could also be offered appropriate training to gain a better understanding of health promotion and its active implementation in everyday work. This should be done in the context of technology use.

### Strengths and limitations

One strength of the study is the use of validated instruments, which have shown strong validity and high internal consistency and could be confirmed in the present study. A further strength of the study was the design of the recruitment, which was based on a clinic register and thus ensured a complete selection and contact with the clinics. Most of the participants were aged 40 years or older. This is in line with current statistics on the age structure of German healthcare workers [56].

A limitation to be considered is that the survey data was collected exclusively on the basis of self-reporting, which can lead to a distortion of the data. In particular, the health-related behaviors of supervisors were assessed by the nursing staff. This assessment may be distorted by their own attitude towards their supervisors. For better validity, both a self-assessment by the supervisor and an external assessment by the nurse should be carried out. The cross-sectional design of the study restricts causal conclusions.

The calculated required sample size of *n* = 273 was not achieved. The reason for this could be the short recruitment phase. Representativeness for all nursing staff in inpatient care cannot be assumed, as it is not possible to speak for all inpatient specialist departments of a hospital. In this study, no differentiation was made according to specialty. In addition, more than half of the participants has a management function in nursing. Geographical representativeness cannot be assumed either, as not all federal states were included in the study.

The response rate could not be clearly determined. In order to ensure data security, the survey didn´t allow a trace back to a specific hospital. That´s why, we aren´t able to say how many nurses from one hospital filled out the survey. In addition, we do not know who of the nursing management contacted actually forwarded the survey to the nursing staff.

A further limitation is to access technostress via an online questionnaire. During the development of the questionnaire, the authors tried to reduce unnecessary information or other aspects that could lead to technostress when filling out the survey. Therefore, the questionnaire could be filled out on a computer and also on smartphone with special settings for better reading.

## Conclusions

Against the background of increasing digitalization in the healthcare sector, it is particularly relevant to gain insights into possible stressors caused by the use of ICT and their health contexts. This cross-sectional study has shown that HoL and technostress are important aspects for the health of nursing staff in inpatient care. However, due to their exploratory nature, these results can only provide an initial indication. The results presented here should be verified in longitudinal studies in order to make causal statements about the influence of technostress and HoL on the health of nursing staff.

## Data Availability

The datasets analyzed during the current study are not publicly available due to German national data protection regulations but are available from the corresponding author on reasonable request.

## Abbreviations

GSA region: Germany, Switzerland, Austria region
HoL: Health-oriented-Leadership
ICT: Information and communication technology
WPC: Work-privacy conflict
